# Pneumatosis Cystoides Intestinalis in Patients with Systemic Sclerosis: A Case Report and Review of 39 Japanese Cases

**DOI:** 10.1155/2016/2474515

**Published:** 2016-08-29

**Authors:** Manabu Kaneko, Shin Sasaki, Shuzo Teruya, Kosuke Ozaki, Kazuhiro Ishimaru, Emi Terai, Hiroshi Nakayama, Toshiyuki Watanabe

**Affiliations:** ^1^Department of Surgery, Omori Red Cross Hospital, 4-30-1 Chuo, Ota-ku, Tokyo 143-8527, Japan; ^2^Department of Internal Medicine, Division of Rheumatology, Omori Red Cross Hospital, 4-30-1 Chuo, Ota-ku, Tokyo 143-8527, Japan

## Abstract

Pneumatosis cystoides intestinalis (PCI) is a rare gastrointestinal complication of systemic sclerosis (SSc) characterized by intramural accumulation of gas within thin-walled cysts. We report the case of an 82-year-old female patient with pneumoperitoneum due to PCI associated with SSc and review the features of the 39 Japanese cases. The median patient age was 57 years (range 24–83 years) and the male/female ratio was 1 : 12. In the recent decade, 14 out of 15 cases (93.3%) evaluated with CT scans were diagnosed with PCI. The results suggest that CT scan may be a useful diagnostic tool for detecting PCI. PCI in patients with SSc is usually benign and requires only conservative therapy. However, two patients (5.1%) with signs of peritoneal irritation required surgery. When peritoneal irritation secondary to additional pathology is observed, surgical treatment may be warranted; a precise diagnosis for this condition is therefore essential.

## 1. Introduction

Pneumatosis cystoides intestinalis (PCI) is a rare gastrointestinal complication of systemic sclerosis (SSc). It is characterized by submucosal and/or subserosal collections of free gas, forming cystic lesions within the gastrointestinal tract and neighboring tissues [1, 2]. This complication is usually asymptomatic and detected incidentally through widespread use of computed tomography (CT) in recent years; however, it can be accompanied by abdominal pain and pneumoperitoneum. Clinical presentations and imaging characteristics in this condition may simulate true gastrointestinal perforation. A precise diagnosis is therefore essential, since PCI is generally treated conservatively. An extensive search on PubMed and the Japanese Medical Abstracts Society found 38 Japanese patients diagnosed with PCI accompanied by SSc over a span of 38 years (from 1978 to 2015). We hereby describe a case of SSc complicated by PCI and review the demographic, clinical, diagnostic, and therapeutic features of the 39 Japanese cases.

## 2. Case Report

**Figure 1:**
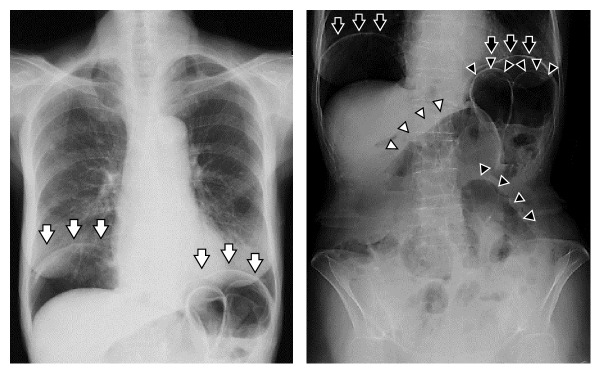
A chest radiograph demonstrating pneumoperitoneum in the subdiaphragmatic regions bilaterally (arrows). A plain abdominal radiograph showing several dilated loops of small intestine (arrowheads).

**Figure 2:**
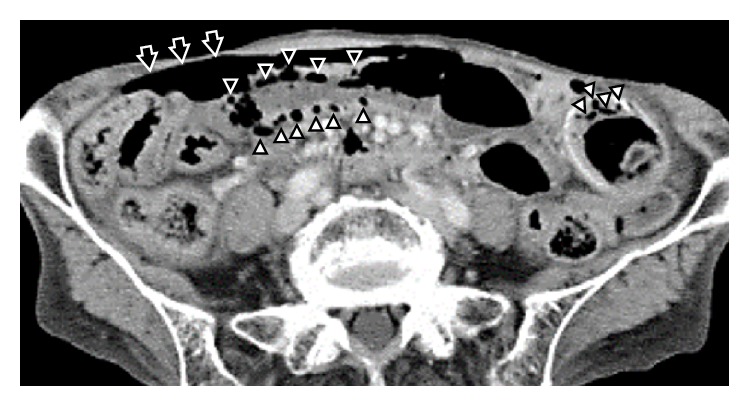
An abdominal computed tomography image demonstrating a large quantity of free air in the abdominal cavity (arrows). Multiple small cysts are seen within the wall of the small bowel (arrowheads), consistent with pneumatosis cystoides intestinalis in this patient with systemic sclerosis.

An 82-year-old woman was referred to our department for incidental finding of pneumoperitoneum on a chest radiograph during a medical checkup at another hospital. Initial history taking revealed a prior surgical history of only a laparotomy secondary to acute appendicitis approximately 60 years before. During physical examination, the patient was afebrile with a pulse rate of 82 beats per minute and blood pressure of 136/86 mmHg. Abdominal examination revealed a slightly distended but soft, tympanic, and nontender abdomen, without signs of peritoneal irritation. There were no palpable masses. Urgent laboratory investigations showed a slightly low hemoglobin level with normal white blood cell count and C-reactive protein level (Table 1). A plain abdominal radiograph showed free air under the diaphragm bilaterally and small bowel dilatation (Figure 1). Abdominal CT scan revealed massive free air and dilated small intestine with gas inside the bowel wall; however, there was no radiological evidence of a localized point of bowel obstruction and no apparent life-threatening acute causes of PCI (Figure 2). Although autoimmune serological testing showed negative results for antinuclear, anti-Scl-70, and anticentromere antibodies, further detailed history taking revealed that the patient had suffered from Raynaud's phenomenon and was previously diagnosed with SSc approximately 30 years before. She recalled that she was treated and followed up for several years, albeit without detailed information regarding her treatment. A rheumatologist later confirmed that she indeed had limited cutaneous SSc, in view of the presence of sclerodactyly of the fingers, telangiectasia on her face, chest, and hands, and Raynaud's phenomenon, all of which met the criteria for SSc [3]. Her pneumoperitoneum was considered to be due to PCI. As the patient was stable with no life-threatening signs and symptoms, she was treated conservatively with bowel rest and maintenance of fluid levels and subsequently discharged home without surgical intervention. The patient remained well eighteen months after discharge, without any abdominal symptoms.

## 3. Discussion

PCI is a rare gastrointestinal complication of SSc characterized by intramural accumulation of gas within thin-walled cysts. Air-filled bubble-like lesions are located in the submucosa or the subserosa of the alimentary tract. Although PCI is most commonly seen in the small intestine, it can also be detected in the large intestine or the stomach [2]. When these air-filled cysts rupture, they cause a pneumoperitoneum, which is generally benign in nature.

We searched on PubMed and the Japanese Medical Abstracts Society for case reports, excluding brief reports on clinical images, containing the key words “pneumatosis cystoides intestinalis” and “systemic sclerosis or scleroderma”. Of note, 39 cases from Japan (including our case) have been reported in the literature. Of these, 35 cases were reported in Japanese, while the others were in English [4–6] (Table 2). Any symptom that was not described was presumed to be absent. Likewise, any test that was not mentioned was presumed to have not been done. The median patient age was 57 years (range 24–83 years) and the male/female ratio was 1 : 12. Of these, 11 patients (28.2%) had SSc of the diffuse type while 23 (59.0%) had the limited type. The remaining 5 patients (12.8%) had types that could not be assessed. The mean disease duration from onset of SSc symptoms to diagnosis of PCI was 6 years (range 0–30 years). Antinuclear, anticentromere, and anti-Scl-70 antibodies were positive in 33, 7, and 3 patients (84.6%, 18.0%, and 7.7%), respectively. Overlap syndrome was seen in 8 patients with polymyositis (20.5%) and 1 patient with Sjogren's syndrome (2.6%). Small intestinal PCI was detected in 29 patients (74.3%) and large intestinal PCI in 4 patients (10.2%). The symptoms observed, in decreasing order, were abdominal distention (*n* = 32, 82.1%), abdominal pain (*n* = 13, 33.3%), abdominal tenderness (*n* = 11, 28.2%), and nausea or vomiting (*n* = 9, 23.1%), among others. Peritoneal irritation was seen in two patients (5.1%), both of whom required surgery. Pneumoperitoneum was involved in 34 patients (87.2%).

Gastrointestinal manifestation in SSc is characterized by atrophy of the muscularis propria and its replacement by collagen tissue, resulting in disturbances of digestive peristalsis and leading to gastroparesis, bacterial overgrowth of the small intestine, or constipation [7]. The etiology of PCI in patients with SSc is not fully understood. However, there are several possible mechanisms of how gas cysts were produced in the bowel wall. The mechanical theory suggests that increased luminal pressure caused by intestinal obstruction allows gas to penetrate into the submucosal space through a mucosal breakdown [8]. The bacterial theory suggests that gas cysts result from either excess hydrogen gas produced by intraluminal bacterial fermentation or altered partial pressure of nitrogen within the intestinal wall [9]. Finally, it has been advocated that long term use of corticosteroids could possibly induce atrophy of the intestinal mucosa, sometimes resulting in a mucosal break and the subsequent translocation of gas into the submucosal layer [10, 11]. In this review, 24 patients (61.5%) were reported to have received corticosteroid therapy.

PCI may be detected through a variety of different radiographic imaging modalities, such as plain radiographs, contrast studies, CT scan, ultrasound, and magnetic resonance imaging. Among these, CT scan is the best diagnostic modality, with better sensitivity than plain radiographs or ultrasound [12]. In actual clinical settings, CT scan should follow a plain radiograph film with suggestive findings such as radiolucent linear or circular air bubbles in the bowel wall with/without subdiaphragmatic free air. In addition, a CT scan can provide additional information that may suggest the existence of other causes of PCI, such as intestinal volvulus, necrosis, or perforation [7]. In this review, CT scan was reported to be useful for detection of PCI in 22 (56.4%) cases. In the recent decade, particularly, 14 out of 15 cases (93.3%) evaluated with CT scans were diagnosed with PCI.

Treatment of PCI should be based on symptoms; in other words, patients with asymptomatic PCI need no treatment. If there are symptoms, excluding peritoneal irritation, conservative treatment, including one or more of the following, may be initiated: fluid and electrolyte supplementation, total parenteral nutrition, bowel rest, bowel tract decompression, elemental diet, antibiotics (tetracycline, metronidazole, or ampicillin), high concentration or hyperbaric oxygen inhalation therapy, and octreotide infusion [13–16]. Antibiotics are used for small intestinal bacterial overgrowth [17]. The bacterial theory is supported by the observation that benign PCI often responds to dietary changes, antibiotic therapy, and oxygen, which is believed to attenuate the gaseous cysts by decreasing the partial pressure of the nitrogenous gases and by being toxic to the anaerobic gut bacteria [9, 18–21]. In this review, the number of patients who received antibiotics, high concentration oxygen therapy, and hyperbaric oxygen therapy was 13 (33.3%), 7 (17.9%), and 2 (5.1%), respectively. Fifteen patients (38.5%) received other treatments such as fluid therapy, bowel tract decompression, or elemental diet. In our case, the patient did not exhibit any signs of peritonitis or bowel ischemia; therefore, conservative management was selected. Surgical treatment is generally not preferred because the disease is almost always generalized, and operative manipulation of a scleroderma bowel tends to result in prolonged ileus [13]. However, emergency surgical exploration must be taken into consideration when the clinical presentation, laboratory results, or imaging tests indicate peritoneal irritation [22]. In this review, two patients (5.1%) with signs of peritoneal irritation required surgery: one for small bowel volvulus and the other for recurrent peritonitis due to inflammation of the dilated jejunal loops resulting from distal jejunal stricture.

In conclusion, PCI among SSc patients is a rare complication, and its pathology is still not fully understood. In most cases, conservative management is preferable to surgical intervention. However, when peritoneal irritation is observed, surgical treatment may be warranted; a precise diagnosis is therefore essential in deciding whether a patient should be subjected to extensive surgery or treated conservatively. CT scan is a useful diagnostic tool for detecting not only PCI, but also additional pathology.

## Figures and Tables

**Table 1:** Laboratory data on admission.

Complete blood count		Blood chemistry		Autoantibody (normal value)	
WBC	4800/*μ*L	TP	7.4 g/dL	Antinuclear (speckled)	1 : 40 (<×40)
RBC	332 × 10^4^/*μ*L	Alb	3.9 g/dL	Anti-Scl-70	8.9 (<10)
Hemoglobin	10.3 g/dL	BUN	20.8 mg/dL	Anticentromere	<5.0 (<10)
Hematocrit	32.1%	Cre	1.0 mg/dL		
Platelet	12.9 × 10^4^/*μ*L	Na	143 mEq/L		
		K	4.4 mEq/L		
		Cl	111 mEq/L		
		T-Bil	0.4 mg/dL		
		AST	20 IU/L		
		ALT	12 IU/L		
		LDH	166 IU/L		
		*γ*-GTP	9 IU/L		
		CRP	0.00 mg/dL		
		HbA1c	5.4%		

WBC: white blood cell; RBC: red blood cell; TP: total protein; Alb: albumin; BUN: blood urea nitrogen; Cre: creatinine; T-Bil: total bilirubin; AST: aspartate aminotransferase; ALT: alanine aminotransferase; LDH: lactate dehydrogenase; *γ*-GTP: *γ*-glutamyl transferase; CRP: C-reactive protein; Na: sodium; K: potassium; Cl: chloride; HbA1c: glycated hemoglobin.

**Table 2:** Demographic data for 39 Japanese cases of SSc with PCI.

Characteristics	Number of patients
Age (years), median (range)	57 (24–83)
Sex	
Female	36 (92.3%)
Male	3 (7.7%)
Types of scleroderma	
Diffuse	11 (28.2%)
Limited	23 (59.0%)
NA	5 (12.8%)
Duration from onset of SSc to onset of PCI (years), median (range)	6 (0–30)
Past corticosteroid therapy	
Present	24 (61.5%)
Autoantibody positivity	
Antinuclear antibody	33 (84.6%)
Anticentromere antibody	7 (18.0%)
Anti-Scl-70 antibody	3 (7.7%)
Negative	6 (15.4%)
Overlap syndrome	
Polymyositis/scleroderma	8 (20.5%)
Sjogren's syndrome/scleroderma	1 (2.6%)
Site of PCI	
Small intestine	27 (69.2%)
Small and large intestine	2 (5.1%)
Large intestine	2 (5.1%)
NA	8 (20.5%)
Symptoms and signs	
Abdominal distention	32 (82.1%)
Abdominal pain	13 (33.3%)
Abdominal tenderness	11 (28.2%)
Nausea/vomiting	9 (23.1%)
Weight loss	8 (20.5%)
Anorexia	8 (20.5%)
Constipation	5 (12.8%)
Fatigue	5 (12.8%)
Diarrhea	5 (12.8%)
Peritoneal irritation sign	2 (5.1%)
Diagnostic modalities	
Upper gastrointestinal series	2 (5.1%)
Plain X-ray film	10 (25.6%)
CT	11 (28.2%)
Plain X-ray film and CT	11 (28.2%)
Exploratory laparotomy	5 (12.8%)
Pneumoperitoneum	
Present	34 (87.2%)
Absent	5 (12.8%)
Treatment	
Antibiotics	13 (33.3%)
Oxygen therapy	9 (23.1%)
Octreotide	2 (5.1%)
Surgery	2 (5.1%)
Treatments other than the above-mentioned ones (symptomatic therapy combined with fluid therapy or elemental diet, etc.)	15 (38.5%)

SSc: systemic sclerosis; PCI: pneumatosis cystoides intestinalis; NA: not available; CT: computed tomography.
